# XRCC1 Arg399Gln was associated with repair capacity for DNA damage induced by occupational chromium exposure

**DOI:** 10.1186/1756-0500-5-263

**Published:** 2012-05-29

**Authors:** Xuhui Zhang, Xuan Zhang, Lei Zhang, Qing Chen, Zhangping Yang, Jingmin Yu, Hong Fu, Yimin Zhu

**Affiliations:** 1 Department of Epidemiology and Biostatistics, Zhejiang University School of Medicine, 388 Yu-Hang-Tang Road, Hangzhou 310058, Zhejiang, People's Republic of China; 2 Hangzhou Center for Disease Control and Prevention, Hangzhou 310021, People's Republic of China; 3 Zhejiang Center for Disease Control and Prevention, Hangzhou 310051, People's Republic of China; 4 Tonglu Center for Disease Control and prevention, Tonglu 311500, People's Republic of China; 5 Jiande Center for Disease Control and prevention, Jiande 311600, People's Republic of China

**Keywords:** DNA damage, Genetic susceptibility, XRCC1, Occupational chromium exposure

## Abstract

**Background:**

Occupational chromium exposure may induce DNA damage and lead to lung cancer and other work-related diseases. DNA repair gene polymorphisms, which may alter the efficiency of DNA repair, thus may contribute to genetic susceptibility of DNA damage. The aim of this study was to test the hypothesis that the genetic variations of 9 major DNA repair genes could modulate the hexavalent chromium (Cr (VI))-induced DNA damage.

**Findings:**

The median (P_25_-P_75_) of Olive tail moment was 0.93 (0.58–1.79) for individuals carrying GG genotype of XRCC1 Arg399Gln (G/A), 0.73 (0.46–1.35) for GA heterozygote and 0.50 (0.43–0.93) for AA genotype. Significant difference was found among the subjects with three different genotypes (*P* = 0.048) after adjusting the confounding factors. The median of Olive tail moment of the subjects carrying A allele (the genotypes of AA and GA) was 0.66 (0.44–1.31), which was significantly lower than that of subjects with GG genotype (*P* = 0.043). The A allele conferred a significantly reduced risk of DNA damage with the OR of 0.39 (95% CI: 0.15–0.99, *P* = 0.048). No significant association was found between the XRCC1Arg194Trp, ERCC1 C8092A, ERCC5 His1104Asp, ERCC6 Gly399Asp, GSTP1 Ile105Val, OGG1 Ser326Cys, XPC Lys939Gln, XPD Lys751Gln and DNA damage.

**Conclusion:**

The polymorphism of Arg399Gln in XRCC1 was associated with the Cr (VI)- induced DNA damage. XRCC1 Arg399Gln may serve as a genetic biomarker of susceptibility for Cr (VI)- induced DNA damage.

## Findings

### Background

Chromium compounds are widely used in industry as chromium compound manufacturing, electroplating, leather tanning, welding, corrosion inhibitors, and alloying metal, etc. The respiratory tract is the major way of occupational exposure to chromium besides oral and dermal exposure. Trivalent chromium(Cr(III)) compound, an essential micronutrient, has been considered to be lower toxicity [1]. Previous studies [2-6] have revealed that hexavalent chromium (Cr(VI)) compounds could induce DNA damage. Exposure to Cr(VI) significantly increases the risk of respiratory tract cancer [7,8], thus Cr(VI) has been classified as a human carcinogen (group 1) by the International Agency for the Research on Cancer (IARC) [9].

Although the mechanisms involved in Cr(VI) induced damage remains to be fully elucidated, generation of reactive oxygen species (ROS) was likely the key step in the intracellular reduction of Cr(VI) by cellular reductant such as reduced glutathione (GSH) and ascorbic acid. The excessive production of ROS may lead to oxidative stress, DNA damage [4,10]. Common forms of DNA damage include DNA strand breaks, chromium-DNA adducts, cross-links and oxidative DNA damage [2-4,11]. However, induced DNA damage could be repaired through individual repair system to maintain the genomic stability. The common mechanisms of DNA repair include base excision repair (BER), nucleotide excision repair (NER), mismatch repair, etc. Therefore, the residue DNA damage, which associates with the risk of lung cancer and other diseases, is dependent on capability of DNA repair. High inter- individual variation of DNA damage had been found in the previous studies on chromium exposure [2,12]. Therefore, the individual DNA repair capacity might modulate the DNA damage and associate with the risk of involved diseases under chromium exposure. DNA repair gene polymorphisms, which may alter the efficiency of DNA repair, contribute to genetic susceptibility of DNA damage. Hence, we hypothesize that the genetic variation of 9 major DNA repair genes could modulated the Cr(VI)- induced damage and might serve as the genetic biomarkers of susceptibility.

To examine this hypothesis, we recruited 157 chromium-exposed electroplating workers and 93 control subjects. DNA damage in peripheral lymphocytes was evaluated with alkaline comet assay. Nine polymorphisms of DNA repair genes XRCC1 Arg399Gln, XRCC1Arg194Trp, ERCC1 C8092A, ERCC5 His1104Asp, ERCC6 Gly399Asp, GSTP1Ile105Val, OGG1 Ser326Cys, XPC Lys939Gln, XPD Lys751Gln were determined. The associations between these polymorphism markers and the Cr (VI)- induced DNA damage were analyzed.

## Materials and Methods

### Study subjects

157 electroplating workers were recruited from 20 electroplating factories in Hangzhou, China from 2009–2010. Control subjects (n = 93) were recruited from the workers without exposure to chromium compounds and other known physical or chemical genotoxic agents. Subjects with abnormal liver or kidney function and suffering from other chronic diseases such as cancers, diabetes, heart diseases were excluded in the study. All the subjects were interviewed for information of age, smoking habits, alcohol consumption, medical history and years of chromium exposure. Short- term sampling of air was conducted according to Specifications of Air Sampling for Hazardous Substances Monitoring in the Workplace in China (GBZ159-2004). Airborne chromium concentration was measured by graphite furnace atomic absorption spectrophotometer (M6 Thermo, USA). About 4 ml of peripheral vein blood sample was collected from each subject and drawn into two vacuum tubes containing 3.6 mg of EDTA. 2 ml of blood was stored at 4°C for determining chromium levels and the comet assay, and the other was stored at −80°C for DNA isolation and genotyping. The study protocol was approved by the Institutional Review Board of Hangzhou Center for Disease Control and Prevention. Written informed consent was obtained from each subject.

### DNA damage determination

The alkaline comet assay was employed to detect the DNA damage in peripheral lymphocytes. The alkaline comet assay was performed as previously described with some modifications [13]. Peripheral blood (10 μl) was mixed with 75 ul of 0.75% low-melting- point agarose and transferred to a microscope slide pre-coated with a layer of 0.75% normal-melting-point agarose. The slides were immersed in the lysis buffer (2.5 mol/L NaCl, 100 mmol/L EDTA, 10 mmol/L Tris, freshly added 1% Triton X-100 and 10% DMSO, pH 10) for 1 h at 4°C to remove proteins. The slides were then placed in a horizontal electrophoresis tank containing electrophoresis buffer (300 mmol/L NaOH, 1 mmol/L EDTA, pH 13) for 20 min to allow DNA unwinding. The electrophoresis was carried out in the same buffer for 20 min. After electrophoresis, the slides were neutralized in the neutralization buffer (0.4 mol/L Tris, pH 7.5), and then stained with 50 μL ethidium bromide solution (20 μg/mL). All the steps were conducted under yellow light to prevent additional DNA damage. One hundred nuclei were selected randomly from each sample. The observation was made at 400× magnification using a fluorescence microscope (DMI 4000) equipped with a 530-nm excitation filter and a computer-based image analysis program CASP. The medians of Olive tail moment, tail length and tail DNA% were used for assessing DNA damage. Olive tail moment is defined as the product of the tail length and the fraction of total DNA in the tail and calculated as [(tail mean - head mean) × (tail DNA%/100)] [13].

### Genotyping

DNA was extracted from peripheral lymphocytes using genomic DNA extraction kits (Tiangen blood genomic DNA extraction kits, Tiangen, China). The genotypes of XRCC1 Arg194Trp and Arg399Gln were determined with PCR-RFLP. The primers of amplification were as follows: XRCC1 Arg194Trp:F: 5′-GCC CCG TCC CAG GTA-3′,R: 5′-AGC CCC AAG ACC CTT TCA CT-3′;XRCC1 Arg399Gln:F: 5′-TTG TGC TTT CTC TGT GTC CA-3′,R: 5′-TCC TCC AGC CTT TTC TGA TA-3′. PCR for XRCC1 Arg194Trp and XRCC1 Arg399Gln were performed under the following conditions: 95°C for 5 min, followed by 30 cycles of 94°C for 40s, 56°C (Arg399Gln) or 61 °C (Arg194Trp) for 30 s and 72°C for 30 s and a final elongation step at 72°C for 10 min. Then the PCR products were digested at 37°C for 12 h with restricted endonucleases MspI (NEB, USA) (Arg399Gln) or PvuII (Arg194Trp). The 10 μL of digested PCR products were electrophoresed on a 2% agarose gel. For Arg399Gln, one band of 615 bp represents gln/gln, two bands (375 bp and 240 bp) represent arg/arg, and three bands (615 bp, 375 bp and 240 bp) represent arg/gln. For Arg194Trp, one band of 491 bp represents arg/arg, two bands (197 bp and 294 bp) represent trp/trp, and three bands (197 bp, 294 bp and 491 bp) represent arg/gln. The genotypes of ERCC1 C8092A, ERCC5 His1104Asp, ERCC6 Gly399Asp, GSTP1Ile105Val, OGG1 Ser326Cys, XPC Lys939Gln, XPD Lys751Gln were detected using TaqMan probe- based real time PCR (7900, ABI, USA). The primer and probe sequences were obtained from the National Cancer Institute’s SNP500Cancer database. Negative controls and previously genotyped samples were included in each plate to ensure the accuracy of the genotyping.

### Statistical analysis

Because distribution of DNA damage data (olive tail moment, tail length and tail DNA%) do not follow normal distribution, the median and P_25_-P_75_ was used to summarize the central location and variation of data, and compared among genotype groups using non-parametric test (Kruskal-Wallis test). Chi square test was used to compare frequency data. A goodness-of-fit Chi square test was used to evaluate for the Hardy-Weinberg equilibrium. Multivariate linear (the DNA damage data was square root-transformed) and logistic models were used to adjust the potential confounding variables including age, gender, smoking status, drinking and occupational exposure time of chromium. A two-sided *P* value of less than 0.05 was considered statistically significant. All statistical calculations were performed by using SPSS 16.0.

## Results

### Characteristics of the study population

The mean age (± standard deviation) of exposed subjects was 39.7 ± 8.3 years while 38.8 ± 9.6 years for control group (*P >* 0.05). There were no significant differences in gender, smoking status, alcohol consumption between the two groups (all *P* values *>* 0.05). The median time of chromium exposure was 5.3 years (range: 0.5 to 23 years).

The median of short-term exposure concentration of chromium in the air at the 20 electroplating workplaces was 0.060 mg/m^3^ (range from 0.016 to 0.531 mg/m^3^), which was higher than the permissible concentration short term exposure limit (PC-STEL) of chromium in China (0.05 mg/m^3^), and 52% factories had chromium level above the standard of PC-STEL.

### The chromium concentration in erythrocytes and DNA damage in exposed and control subjects

Chromium concentrations in erythrocytes and DNA damage in exposed and control groups were shown in Table 1. The exposed subjects had the median (P_25_-P_75_) of chromium concentration in erythrocytes of 4.41 μg/l (2.50, 5.29), which was about two times higher than that in control subjects (1.54 μg/l (0.61–2.98), *P* < 0.001). The medians of Olive tail moment, tail length and tail DNA% in exposed group were 1.13(0.47–1.45), 11.77 (6.42–14.84) and 3.69 (2.50–5.29), respectively, and were significantly higher than those in control subjects (0.12 (0.04–0.22), 3.26 (3.00–4.00) and 0.59 (0.19–1.11) (*P* < 0.001).

**Table 1:** The chromium concentration in erythrocytes (μg/l) and DNA damage in exposed and control subjects

	**Exposed subjects n = 149**	**Control subjects n = 77**	***P value***^a^
chromium concentration in erythrocytes (μg/l)	4.41 (2.50–5.29)	1.54 (0.61–2.98)	<0.001
Olive tail moment	1.13 (0.47–1.45)	0.12 (0.04–0.22)	<0.001
Tail Length	11.77 (6.42–14.84)	3.26 (3.00–4.00)	<0.001
Tail DNA%	3.69 (2.50–5.29)	0.59 (0.19–1.11)	<0.001

1. Data were presented as median (P_25_-P_75_).

2. *P* values were calculated with rank sum test between the exposed and control subjects.

3. Owing to missing detect in DNA damage and genotype, the numbers of subjects in Table 2 were less 157 in exposed group and 93 in control group.

### Genotypes of the polymorphism of XRCC1 Arg399Gln and the DNA damage induced by Cr (VI) exposure

The allele frequencies for XRCC1 399Gln, XRCC1 194Trp, OGG1 326Cys, ERCC1 8092A, ERCC5 1104Asp, ERCC6 399Asp, XPD 751Gln, XPC 939Gln, GSTP1105Val were 0.24, 0.24, 0.54, 0.57, 0.49, 0.46, 0.08, 0.38, 0.16, respectively. Genotype distributions of all the polymorphisms studied were all consistent with Hardy-Weinberg equilibrium (*P* > 0.05). The medians and P_25_-P_75_ of Olive tail moment, tail length and tail DNA% in the subjects with different genotypes were shown in Table 2.

**Table 2:** DNA damage in the exposed subjects with different genotypes of polymorphisms

**SNP**	**genotype**	**n**	**Olive tail moment**		**Tail length**		**Tail DNA%**	
			**Median**	**P**_**25**_**-P**_**75**_	**Median**	**P**_**25**_**-P**_**75**_	**Median**	**P**_**25**_**-P**_**75**_
XRCC1 Arg399Gln	GG	70	0.93	0.58–1.79	9.74	6.89–18.14	3.25	1.94–5.58
	GA	42	0.73	0.46–1.35	8.19	5.93–13.79	2.50	1.77–4.36
	AA	8	0.50	0.43–0.93	6.64	5.00–9.92	1.91	1.43–3.03
	P^*^		0.048		0.050		0.039	
	AA + GA	50	0.66	0.44–1.31	8.00	5.69–13.74	2.32	1.66–4.33
	*P*^∮^		0.043		0.067		0.046	
XRCC1 Arg194Trp	CC	66	0.75	0.47–1.45	8.55	6.30–14.22	2.82	1.66–4.85
	CT	49	0.91	0.48–1.49	9.69	6.97–16.25	3.22	1.83–4.75
	TT	5	0.66	0.37–1.82	7.67	5.11–16.94	2.39	1.29–5.84
	P^*^		0.973		0.964		0.992	
OGG1 Ser326Cys	CC	18	1.09	0.41–1.84	11.72	4.92–18.87	3.63	1.55–5.80
	CG	56	0.86	0.47–1.46	9.18	6.50–15.53	3.07	1.73–4.86
	GG	41	0.78	0.56–1.19	8.93	6.87–12.07	2.69	2.02–4.15
	P^*^		0.462		0.335		0.377	
ERCC1 C8092A	CC	20	0.84	0.45–1.38	9.79	6.56–15.53	3.20	1.77–4.72
	CA	47	0.71	0.47–1.43	8.10	5.81–14.10	2.45	1.61–4.53
	AA	49	0.92	0.62–1.60	9.66	7.21–17.29	3.22	2.06–5.50
	P^*^		0.543		0.690		0.595	
ERCC5 His1104Asp	GG	25	0.92	0.62–1.60	9.66	7.21–17.29	3.22	2.06–5.50
	GC	61	0.84	0.54–1.50	9.66	6.79–15.88	2.84	1.99–4.99
	CC	30	0.83	0.50–1.38	9.21	7.01–15.05	3.04	1.81–4.59
	P^*^		0.886		0.931		0.920	
ERCC6 Gly399Asp	CC	32	0.83	0.49–1.14	8.90	6.82–11.70	3.03	1.87–3.97
	CT	54	0.75	0.48–1.73	8.26	6.42–16.12	2.75	1.85–5.51
	TT	31	1.03	0.48–1.57	10.70	5.72–18.10	3.22	1.68–5.19
	P^*^		0.143		0.152		0.203	
XPD Lys751Gln	AA	94	0.82	0.48–1.45	9.50	6.42–14.84	2.94	1.81–4.80
	AC	20	0.81	0.48–1.81	8.79	7.00–16.74	2.88	1.88–5.81
	CC	0						
	P^*^		0.771		0.742		0.900	
XPC Lys939Gln	GG	45	0.89	0.56–1.52	9.66	6.97–15.96	2.96	2.02–5.14
	GT	53	0.83	0.47–1.40	8.87	6.63–14.07	2.75	1.39–6.13
	TT	18	0.81	0.39–2.10	9.13	5.68–23.00	2.92	1.73–4.57
	P^*^		0.564		0.379		0.705	
GSTP1 Ile105Val	AA	80	0.83	0.52–1.58	9.21	6.75–15.65	2.94	1.85–5.30
	AG	30	0.70	0.38–1.41	8.03	5.71–15.29	2.41	1.62–4.53
	GG	6	1.02	0.71–1.84	11.89	8.83–18.95	3.74	2.46–5.83
	P^*^		0.169		0.514		0.463	

* The DNA damage data was square root-transformed and multivariable linear model was used after adjusting confounding factors of gender, age, smoking status, drinking and exposure time of chromium.

Comparing the subjects with GG genotype with AA + GA.

∫ Owing to missing detect in DNA damage and genotype, the numbers of subjects in Table 2 were less 157 in exposed group.

The median of Olive tail moment (P_25_–P_75_) was 0.93(0.58–1.79) for individuals carrying GG genotype of XRCC1 Arg399Gln (G/A), 0.73 (0.46–1.35) for GA heterozygote and 0.50 (0.43–0.93) for AA genotype, respectively. After adjusting the confounding factors of gender, smoking status, drinking and occupational exposure time of chromium, significant difference in Olive tail moment was found among the subjects with different genotypes (*P* = 0.048). The median of Olive tail moment of the subjects carrying A allele (the genotypes of AA and GA) was 0.66 (0.44–1.31), which was significantly lower than that for those with GG genotype (*P* = 0.043).

Similar results were observed for tail length and tail DNA% in different groups. After adjustment of the potential confounding factors, significant differences were found in tail length (*P* = 0.050) and tail DNA% (*P* = 0.039) among different genotypes. The medians of tail length were 9.74 (6.89–18.14) for individuals carrying GG genotype in XRCC1 Arg399Gln (G/A), 8.19 (5.93–13.79) for GA heterozygote and 6.64 (5.00–9.92) for AA genotype, respectively. The medians of tail DNA% were 3.25 (1.94–5.58) for individuals carrying GG genotype of XRCC1 Arg399Gln (G/A), 2.50 (1.77–4.36) for GA heterozygote and 1.91 (1.43–3.03) for AA genotype, respectively. The median of tail DNA% of the subjects with carrying A allele (the genotypes of AA and GA) was 2.32 (1.66–4.33), which was significantly lower than that for those with GG genotype (*P* = 0.046). A borderline statistical difference was found in tail length when compared the subjects carrying A allele with GG genotype (*P* = 0.067).

No statistical association in Olive tail moment, tail length and tail DNA% was found among different genotypes of XRCC1 Arg194Trp, ERCC1 C8092A, ERCC5 His1104Asp, ERCC6 Gly399Asp, GSTP1 Ile105Val, OGG1 Ser326Cys, XPC Lys939Gln, XPD Lys751Gln (all the *P* value >0.05).

**Figure 1:**
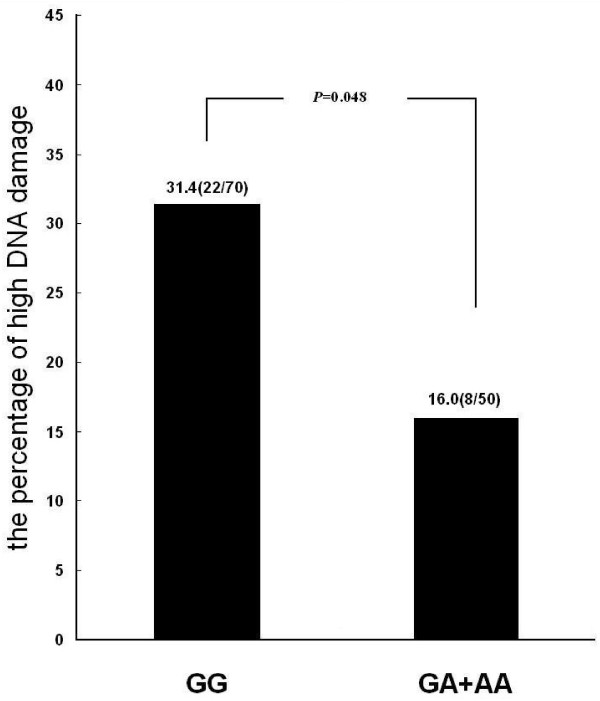
**The percentage of high DNA damage in different genotypes of XRCC1 399.** DNA damage was quantitatively assessed with Olive tail moment by alkaline comet assay. High DNA damage was defined as great than the value (1.44) of percentile 75 of Olive tail moment.

With the 75th percentile of Olive tail moment (1.44) as a cut-off point, the subjects were divided into two groups: high DNA damage (>1.44) and low DNA damage (≦1.44). 31.4% (22/70) of the subjects carrying GG genotype of XRCC1 Arg399Gln (G/A) had higher DNA damage (>1.44 of olive tail moment) while only 16.0% (8/50) in the subjects carrying A allele. Dose- response relationship was found between the number of A allele and DNA damage (*P*_trend adjusted_ = 0.031). Comparing with the subjects with genotypes of GG, the subject carrying A allele was significantly associated with the reduced risk of DNA damage with the odds ratio of 0.388 (95% CI: 0.152–0.992, *P* = 0.048) after adjusting the potential confounders of gender, smoking status, drinking and exposure time of chromium (Figure 1).

## Discussion

In the present study, we found the chromium concentration in erythrocytes was found to be significantly higher in electroplating workers (4.41 μg/l) than that in control subjects. The finding indicated there was hexavalent chromium exposure in electroplating workplace. Occupational chromium exposure in electroplating induced DNA damage. We also found that the polymorphisms of XRCC1 Arg399Gln was associated with Cr(VI)- induced DNA damage. Our findings supported the hypothesis that the genetic variation of major DNA repair genes could modulate the Cr (VI)- induced damage. The DNA repair capacity may associate with the risk of chromium exposure induced disease such as lung cancer and XRCC1 Arg399Gln could be served as a genetic biomarker of susceptibility for chromium exposure.

Cr (VI) compound can actively enter into the cells through the isoelectric and isostructural anion channels [5], phagocytosis [14], et al. Once inside the cell and in the presence of cellular reductants, such as ascorbate and thiols, Cr (VI) compounds can be reduced through short-lived Cr intermediates (Cr (V) and Cr (IV)) to the stable trivalent state Cr (III) [15]. During these reactive processes, reactive oxygen species (ROS) such as hydroxyl radicals, single oxygen, superoxide and hydrogen peroxide, were generated. The resulting excessive production of ROS may lead to oxidative damage, DNA adducts and crosslinks [16,17]. Iarmarcovai et al. [18]. found the binucleated micro-nucleated cell rate in chromium-exposed welding worker was significantly higher than in control subjects. In the previous study [2], we found the Cr (VI) exposed electroplating workers had higher concentrations of 8-OHdG (an indicator of oxidative DNA damage), olive tail moment, tail length and tail DNA% which were evaluated by comet assay. These findings were in agreement of the other previous studies [3,19]. Therefore, Cr (VI) is a genotoxic agent and associated the risk of lung cancer and other occupational diseases [15].

The DNA repair mechanisms are responsible for repairing the xenobiotic induced DNA damage and maintaining the genomic stability. DNA repair system is involved in the repair of Cr (VI)- induced DNA lesion such as Oxidative damage and single strand break, which are the main forms of DNA damage. Base excision repair (BER) pathway is mainly responsible for repair these DNA lesions. X-ray repair cross-complementing group 1(XRCC1) is a key component in repairing both direct SSB and indirect SSB generated indirectly during base excision repair [20]. It serves as a scaffold connecting many of the other proteins involved in SSB repair. XRCC1 is recruited to SSBs by poly(ADP-ribose)polymerase (PARP1) and then interacts with a number of important proteins involved in SSB such as proliferating cell nuclear antigen (PCNA), DNA polymerase beta (Polb), and polynucleotide kinase (PNK) [20,21]. XRCC1 was essential to reduce the formation of lead chromate induced chromatid lesions [22]. XRCC1 protein is involved in the repair of Cr(VI)-induced SSB [23] and in protection from lead chromate–induced chromosome instability [22].

Genetic polymorphisms of key proteins might be involved in inter-individual variations of DNA repair processes and influence the extent of genotoxic damage. XRCC1 Arg399Gln, a functional genetic variation, may alter the capacity of XRCC1 to interact with several acting enzymes and associated with the efficiency of BER repair [24,25]. Many previous studies [26-28] revealed XRCC1 Arg399Gln was associated with the common cancers such as lung, bladder, esophageal cancers, etc. and modulated the cancer risk for common environmental exposure. In chromium-exposed electroplating workers, we found the polymorphism of XRCC1 Arg399Gln was associated the Olive tail moment, tail length and tail DNA%. The subject carrying A allele reduced the risk of high DNA damage. In the previous studies, XRCC1 Arg399Gln was also found to be associated with chromosome aberrations [29] and micronuclei [18,30] in the chromium exposed welding worker. This polymorphism was also found to modulate the risk of DNA damage induced other occupational exposure to DNA damage agents such as vinyl chloride [31], asbestos [32] and 1,3-butadiene [33].

However, we didn’t find significant association between XRCC1 Arg194Trp and the Cr (VI)-induced DNA damage. This finding was inconsistent with the previous studies [31,33]. We also failed to find any associations of the polymorphisms of ERCC1 C8092A, ERCC5 His1104Asp, ERCC6 Gly399Asp, GSTP1 Ile105Val, OGG1 Ser326Cys, XPC Lys939Gln, XPD Lys751Gln with the DNA lesions. One of the possible reasons might be the specific effect of the DNA repair proteins responding to Cr (VI)-induced DNA damage. The other might be small sample size and relative low statistical power of test in this study. Only 122 chromium exposed electroplating workers were studied and false negative results might exist. Therefore, further study is required with large sample size and more statistical power to screen new genetic biomarkers of Cr (VI)-induced DNA damage.

In conclusion, we found that the genetic variation of XRCC1 Arg399Gln was associated with the Cr (VI)-induced DNA damage. XRCC1 Arg399Gln may be served as a genetic marker of susceptibility and high risk individual identification for chromium exposed workers. With this genetic biomarker, the susceptible population could be screened and prevent from chromium exposure.

## Competing interests

The authors declare that they have no competing interests.

## Authors’ contributions

XZ participated in the epidemiological investigation, performed comet assay and ELISA, analyzed data and drafted the manuscript. X-HZ was a chief investigator and was responsible for the epidemiological design, and sample collection and drafting the manuscript. X-HZ and XZ contributed equally to this work. LZ, Z-PY, HF, J-MY and QC carried out the health surveillance in the workplace and sample collection. Y-MZ participated in the overall design, study coordination, data analysis, and finalized the draft of the manuscript. All the authors read and approved the final manuscript.

## References

[B1] Eastmond DA Macgregor JT Slesinski RS Trivalent chromium: assessing the genotoxic risk of an essential trace element and widely used human and animal nutritional supplement Crit Rev Toxicol 2008 38 3 173 190 10.1080/10408440701845401 18324515

[B2] Zhang XH Zhang X Wang XC Jin LF Yang ZP Jiang CX Chen Q Ren XB Cao JZ Wang Q Chronic occupational exposure to hexavalent chromium causes DNA damage in electroplating workers BMC Public Health 2011 11 1 224 10.1186/1471-2458-11-224 21481275 PMC3094242

[B3] Kuo HW Chang SF Wu KY Wu FY Chromium(VI) induced oxidative damage to DNA: increase of urinary 8-hydroxydeoxyguanosine concentrations (8-OHdG) among electroplating workers Occupational and Environmental Medicine 2003 60 8 590 594 10.1136/oem.60.8.590 12883020 PMC1740592

[B4] Patlolla AK Barnes C Yedjou C Velma VR Tchounwou PB Oxidative stress, DNA damage, and antioxidant enzyme activity induced by hexavalent chromium in Sprague–Dawley rats Environ Toxicol 2009 24 1 66 73 10.1002/tox.20395 18508361 PMC2769560

[B5] Chiu A Katz AJ Beaubier J Chiu N Shi X Genetic and cellular mechanisms in chromium and nickel carcinogenesis considering epidemiologic findings Mol Cell Biochem 2004 255 1–2 181 194 14971659 10.1023/b:mcbi.0000007274.25052.82

[B6] Chiu A Shi XL Lee WK Hill R Wakeman TP Katz A Xu B Dalal NS Robertson JD Chen C Review of chromium (VI) apoptosis, cell-cycle-arrest, and carcinogenesis J Environ Sci Health C Environ Carcinog Ecotoxicol Rev 2010 28 3 188 230 10.1080/10590501.2010.504980 20859824 PMC4330561

[B7] Davies JM Easton DF Bidstrup PL Mortality from respiratory cancer and other causes in United Kingdom chromate production workers Br J Ind Med 1991 48 5 299 313 2039742 10.1136/oem.48.5.299 PMC1012038

[B8] De Flora S Threshold mechanisms and site specificity in chromium(VI) carcinogenesis Carcinogenesis 2000 21 4 533 541 10.1093/carcin/21.4.533 10753182

[B9] IARC Chromium, nickel and welding IARC Monogr Eval Carcinog Risks Hum 1990 49 1 648 2232124 PMC7681426

[B10] Nordberg J Arner ES Reactive oxygen species, antioxidants, and the mammalian thioredoxin system Free Radic Biol Med 2001 31 11 1287 1312 10.1016/S0891-5849(01)00724-9 11728801

[B11] Wang XF Xing ML Shen Y Zhu X Xu LH Oral administration of Cr(VI) induced oxidative stress, DNA damage and apoptotic cell death in mice Toxicology 2006 228 1 16 23 10.1016/j.tox.2006.08.005 16979809

[B12] Lee AJ Hodges NJ Chipman JK Interindividual variability in response to sodium dichromate-induced oxidative DNA damage: role of the Ser326Cys polymorphism in the DNA-repair protein of 8-oxo-7,8-dihydro-2′-deoxyguanosine DNA glycosylase 1 Cancer Epidemiol Biomarkers Prev 2005 14 2 497 505 10.1158/1055-9965.EPI-04-0295 15734978

[B13] Singh NP McCoy MT Tice RR Schneider EL A simple technique for quantitation of low levels of DNA damage in individual cells Exp Cell Res 1988 175 1 184 191 10.1016/0014-4827(88)90265-0 3345800

[B14] Codd R Dillon C Levina A Lay P Studies on the genotoxicity of chromium: from the test tube to the cell Coordination Chemistry Reviews 2001 216 537 582

[B15] Valko M Morris H Cronin M Metals, toxicity and oxidative stress Current Medicinal Chemistry 2005 12 10 1161 1208 10.2174/0929867053764635 15892631

[B16] Gao M Binks S Chipman J Levy L Braithwaite R Brown S Induction of DNA strand breaks in peripheral lymphocytes by soluble chromium compounds Human & Experimental Toxicology 1992 11 2 77 82 10.1177/096032719201100203 1349223

[B17] Macfie A Hagan E Zhitkovich A Mechanism of DNA-Protein Cross-Linking by Chromium Chemical Research in Toxicology 2010 23 2 341 347 10.1021/tx9003402 19877617 PMC2822107

[B18] Iarmarcovai G Sari-Minodier I Chaspoul F Botta C De Meo M Orsiere T Berge-Lefranc J Gallice P Botta A Risk assessment of welders using analysis of eight metals by ICP-MS in blood and urine and DNA damage evaluation by the comet and micronucleus assays; influence of XRCC1 and XRCC3 polymorphisms Mutagenesis 2005 20 6 425 432 10.1093/mutage/gei058 16234265

[B19] Gambelunghe A Piccinini R Ambrogi M Villarini M Moretti M Marchetti C Abbritti G Muzi G Primary DNA damage in chrome-plating workers Toxicology 2003 188 2 187 195 10.1016/S0300-483X(03)00088-X 12767690

[B20] Caldecott KW XRCC1 and DNA strand break repair DNA Repair (Amst) 2003 2 9 955 969 10.1016/S1568-7864(03)00118-6 12967653

[B21] Fan R Kumaravel TS Jalali F Marrano P Squire JA Bristow RG Defective DNA strand break repair after DNA damage in prostate cancer cells: implications for genetic instability and prostate cancer progression Cancer Res 2004 64 23 8526 8533 10.1158/0008-5472.CAN-04-1601 15574758

[B22] Grlickova-Duzevik E Wise SS Munroe RC Thompson WD Wise JP XRCC1 protects against particulate chromate-induced chromosome damage and cytotoxicity in Chinese hamster ovary cells Toxicol Sci 2006 92 2 409 415 10.1093/toxsci/kfl021 16714390

[B23] Christie NT Cantoni O Evans RM Meyn RE Costa M Use of mammalian DNA repair-deficient mutants to assess the effects of toxic metal compounds on DNA Biochem Pharmacol 1984 33 10 1661 1670 10.1016/0006-2952(84)90289-2 6233980

[B24] Qu T Morii E Oboki K Lu Y Morimoto K Micronuclei in EM9 cells expressing polymorphic forms of human XRCC1 Cancer Lett 2005 221 1 91 95 10.1016/j.canlet.2004.08.013 15797631

[B25] Marsin S Vidal AE Sossou M Ménissier-de Murcia J Le Page F Boiteux S de Murcia G Radicella JP Role of XRCC1 in the coordination and stimulation of oxidative DNA damage repair initiated by the DNA glycosylase hOGG1 J Biol Chem 2003 278 45 44068 44074 10.1074/jbc.M306160200 12933815

[B26] Kim IS Lee GW Kim DC Kim HG Kim S Oh SY Kim SH Kwon HC Polymorphisms and haplotypes in the XRCC1 gene and the risk of advanced non-small cell lung cancer J Thorac Oncol 2010 5 12 1912 1921 10.1097/JTO.0b013e3181f46708 20978448

[B27] Hodgson ME Poole C Olshan AF North KE Zeng D Millikan RC Smoking and selected DNA repair gene polymorphisms in controls: systematic review and meta-analysis Cancer Epidemiol Biomarkers Prev 2010 19 12 3055 3086 10.1158/1055-9965.EPI-10-0877 20935063 PMC3108462

[B28] Huang Y Li L Yu L XRCC1 Arg399Gln, Arg194Trp and Arg280His polymorphisms in breast cancer risk: a meta-analysis Mutagenesis 2009 24 4 331 339 10.1093/mutage/gep013 19465687

[B29] Halasova E Matakova T Musak L Polakova V Letkova L Dobrota D Vodicka P Evaluating chromosomal damage in workers exposed to hexavalent chromium and the modulating role of polymorphisms of DNA repair genes Int Arch Occup Environ Health 2011 10.1007/s00420-011-0684-x 21858514

[B30] Iarmarcovai G Sari-Minodier I Orsière T De Méo M Gallice P Bideau C Iniesta D Pompili J Bergé-Lefranc JL Botta A A combined analysis of XRCC1, XRCC3, GSTM1 and GSTT1 polymorphisms and centromere content of micronuclei in welders Mutagenesis 2006 21 2 159 165 10.1093/mutage/gel010 16551674

[B31] Qiu YL Wang W Wang T Sun P Wu F Zhu SM Qian J Jin L Au W Xia ZL DNA repair gene polymorphisms and micronucleus frequencies in Chinese workers exposed to vinyl chloride monomer Int J Hyg Environ Health 2011 214 3 225 230 10.1016/j.ijheh.2010.12.001 21216194

[B32] Zhao XH Jia G Liu YQ Liu SW Yan L Jin Y Liu N Association between polymorphisms of DNA repair gene XRCC1 and DNA damage in asbestos-exposed workers Biomed Environ Sci 2006 19 3 232 238 16944782

[B33] Wang Q Wang AH Tan HS Feng NN Ye YJ Feng XQ Liu G Zheng YX Xia ZL Genetic polymorphisms of DNA repair genes and chromosomal damage in workers exposed to 1,3-butadiene Carcinogenesis 2010 31 5 858 863 10.1093/carcin/bgq049 20223788

